# Solanum Fruits: Phytochemicals, Bioaccessibility and Bioavailability, and Their Relationship With Their Health-Promoting Effects

**DOI:** 10.3389/fnut.2021.790582

**Published:** 2021-11-25

**Authors:** Cristina Alicia Elizalde-Romero, Luis Aurelio Montoya-Inzunza, Laura Aracely Contreras-Angulo, J. Basilio Heredia, Erick Paul Gutiérrez-Grijalva

**Affiliations:** ^1^Centro de Investigación en Alimentación y Desarrollo, Culiacán, Mexico; ^2^Cátedras CONACYT-Centro de Investigación en Alimentación y Desarrollo, Culiacán, Mexico

**Keywords:** solanum, antioxidant, bioactive, bioaccessibility, bioavailability, eggplant

## Abstract

The *Solanum* genus is the largest in the Solanaceae family containing around 2,000 species. There is a great number of edibles obtained from this genus, and globally, the most common are tomato (*S. lycopersicum*), potato (*S. tuberosum*), and eggplant (*S. melongena*). Other fruits are common in specific regions and countries, for instance, *S. nigrum, S. torvum, S. betaceum, and S. stramonifolium*. Various reports have shown that flavonoids, phenolic acids, alkaloids, saponins, and other molecules can be found in these plants. These molecules are associated with various health-promoting properties against many non-communicable diseases, the main causes of death globally. Nonetheless, the transformations of the structure of antioxidants caused by cooking methods and gastrointestinal digestion impact their potential benefits and must be considered. This review provides information about antioxidant compounds, their bioaccessibility and bioavailability, and their health-promoting effects. Bioaccessibility and bioavailability studies must be considered when evaluating the bioactive properties of health-promoting molecules like those from the Solanum genus.

## Introduction

The numerous species of the *Solanum* genus are distributed mainly in tropical and subtropical areas around the globe; these are used in folk medicine or food crops. The positive effects on the human health of these plants are linked to their content of phenols, alkaloids, saponins, terpenes, flavonoids, coumarins, and carotenoids (1, 2). Some have been reported with anticancer, antioxidant, antidepressant, antihypertensive, anti-inflammatory, hypolipidemic, hypoglycemic, hepatoprotective, anti-obesogenic, and antidiabetic properties (1–4).

In addition, there are reports regarding the bioaccessibility and bioavailability of these molecules. Bioactive compounds are subjected to modifications during processing and gastrointestinal digestion. Moreover, those that permeate the intestinal barrier are metabolized, and then most are distributed for excretion. Thus, the bioavailability of bioactive molecules is often low (5). The low bioavailability of bioactive compounds can hinder their potential bioactive effects on human health. Thus, they are important factors to consider during *in vitro* and *in vivo* evaluations. The present document focuses on consumables matrices and those with potential bioactive effects. A summary of the compounds identified and isolated from *Solanum* species and their potential bioactive properties can be found in Table 1.

**Table 1:** Phytochemicals of *Solanum* species and their biological effects.

***Solanum* species**	**Part**	**Isolated compound**	**Metabolite concentration**	**Effect**	**References**
*S. melongena*	Root	Cannabisin D Cannabisin F Cannabisin G Grossamide Melongenamide B Melongenamide C Melongenamide D	IC_50_ values: 5.1.1 μM 16.2 μM 50.5 μM 26 μM 44.4 μM 16.4 μM 58.5 μM	Anti-inflammatory: reduced NO production in LPS-stimulated RAW 264.7 macrophages	(6)
		N-trans-Feruloyltyramine	IC_50_ value: 5.3 μM	Anti-diabetic: inhibits enzyme α-glucosidase.	(7)
	Peel	Isolated saponins (no specified)	1.2–9.9 mg	Anti-obesogenic: inhibits enzyme pancreatic lipase.	(8)
*S. torvum*	Aerial	Torvoside M	IC_50_ value: 25.2–34.2 μg/mL	Anti-cancer: cytotoxic activity against cancer cell lines MGC-803, HepG2, A549, and MCF-7.	(9)
		Neochlorogenin 6-O-[β-D-xylopyranosyl-(1 → 3)-β-D- quinovopyranoside]	IC_50_ Value: 2.87 μM	Anti-neutrophilic inflammatory: reduced O^2^- generation.	(10, 11)
	Fruit	25(S)-26-O-β-D-glucopyranosyl-5αfurost-22(12)-en-3β,6α,26-triol-6-O-[α-L-rhamnopyranosyl-(1 → 3)-O-β-D-quinovopyranoside] 25(S)-26-O-β-D-glucopyranosyl-5α-furost-22(12)-en-3-one-6α,26-diol-6-O-[α-L-rhamnopyranosyl- (1 → 3)-O-β-D-quinovopyranoside] 25(S)-26-O-β-D-glucopyranosyl-5α-furost-22(12)-en3β,6α,26-triol-6-O-β-D-quinovopyranoside 5α-pregn-16-en-20-one-3β,6α-diol-6-O-[α-Lrhamnopyranosyl-(1 → 3)-β-D-quinovopyranoside]	IC_50_ values: 30–260 μM	Anti-cancer: cytotoxic activity against human melanoma cell lines.	(13)
	Root	Torvanol A	IC_50_ value: 9.6 μg/mL	Antiviral: activity against herpes simplex virus type 1 (mechanism not specified)	(14, 15)
	Seeds	Tovanol A	10 and 30 mg/kg	Antidepressant and anxiolytic: modulation of the noradrenergic, dopaminergic, serotonergic and gabaergic mechanisms.	
	Fruit	Methyl cafeate	3 mg/mL	Antiproliferative: Increased activity of caspases-3 and PARP. Bcl-2 protein is down regulated; Bid and Bax are up regulated.	(16–20)
*S. cernuum*	Leaf	Cycloeucalenone, 24-oxo-31-norcycloartanone	300 and 600 mg/kg	Analgesic and anti-inflammatory: Produced inhibition on writhing by acetic acid.	(21)
*S. lyratum*	Whole	Solajiangxin A-C	ED_50_ value: 1.9–3-7 μg/mL	Anti-cancer: cytotoxicity against mouse lymphocytic leukemia, human nasopharyngeal and human colon adenocarcinoma.	(22)
*S. septemlobum*	Whole	Septemlobin A-C	IC_50_ value: 3.8–7.5 μM	Anti-cancer: inhibited growth of three cancer cell lines (P-388, HONE-1, HT-29)	(23)
*S. anguvi*	Fruit	Gallic acid Chlorogenic acid Caffeic acid Rutin Quercetine	17.54 mg/g 21.90 mg/g 16.64 mg/g 14.71 mg/g 7.39 mg/g	Antioxidant. Free radical scavenging, iron-chelation activity. Inhibit cerebral and hepatic lipid peroxidation.	(24)
*S. betaceum*	Fruit	Keracyanin, Pelargonidin 3-rutinoside, Tulipanin, Delphinidin 3-O-α-l-rhamnosyl-(1–5, 25)-β-dglucoside-3′-O-β-d-glucoside	Not specified	Antioxidant. Capabilty of capturing free radicals due to the presence of hydroxyl groups in the *ortho* position.	(26, 27)
*S. elaeagnifolium*	Aerial	Kaempferol 8-C-β-d-galactoside	25–75 g/kg	Hepatoprotective. Curative effect against histopathological and histochemical damage in liver. Aminorates elevation of aminotranferases.	(28)
*S. americanum*	Aerial	N-trans-pcoumaroyloctopamine, N-trans-p-coumaroyltyramine	IC_50_ values: 2.3 μM 2.7 μM	Anti-diabetic: inhibits enzyme α-glucosidase.	(29)
*S. capsicoides*	Seed	Carpesterol	GI_50_ values: 24–32 μg/mL	Antiproliferative: Inhibits proliferation of human cancer cell lines (glioma, breast, kidney, ovary and erithrolukemic).	(30)

### Phenolic Compounds

Phenolics have at least one aromatic ring with one hydroxyl group in their structure. Phenolics can be classified into flavonoids and non-flavonoids (25). One of the most common species worldwide is *S. lycopersicum*, which is reported with large concentrations of phenolic like chlorogenic acid, resveratrol, quercetin, and myricetin (31–33). Moreover, *S. tuberosum*, has been reported with high concentrations of phenolics in the peels, mainly phenolic acids, especially chlorogenic acid (34). Furthermore, *S. melongena*, where hydroxycinnamic acid derivatives are reported as major phenolics (35). Also, *S. nigrum* had as major compounds myricetin, 3,4-dicaffeoylquinic acid, 3-caffeoylquinic acid, 5-caffeoylquinic acid, and 4,5-dicaffeoylquinic (36). *S. betaceum* had chlorogenic acid and 3-O-caffeoylquinic acid as major compounds (37). The *S. stramonifolium* plant reports high concentrations of phenolics, and the root extract presents anticancer effects (38). Less common species such as *S. scabrum* and *S. burbankii* have the presence of petunidin, delphinidin, and malvidin (39). Some health-related effects of the main phenolics present in this genus are antioxidant, anti-inflammatory, antidiabetic, cardioprotective, and anti-obesity. Phenolic compounds from diverse sources are highly unstable during the gastrointestinal digestion and have low bioavailability (3, 40).

### Alkaloids

Alkaloids are compounds with at least one nitrogen atom in their structure. They can be classified according to their origin, chemical characteristics, or depending on the biosynthetic pathway from which they were derived. However, we can mostly find them classified as true alkaloids, protoalkaloids, and pseudoalkaloids (41). The genus *Solanum* is within the Solanaceae family and is recognized for its alkaloid content and anti-proliferative effects. Other health-promoting effects attributed to alkaloids are antioxidant, antidiabetic; they are considered potential drugs for treating neurodegenerative disorders such as Huntington disease, Parkinson's disease, epilepsy, schizophrenia, and Alzheimer's disease (2, 42, 43). In *S. tuberosum*, dehydrochaconine, chaconine isomers, α-chaconine, solanidadienol chacotriose, solanidadiene solatriose, solanidenol chacotriose, α-solanine, leptine II, α- solanine, α-chaconine, dehydrocommersonine, commersonine, demissine, sisunine, are distributed in different parts of the plant (12, 44–48). Likewise, in *S. lycopersicum*, the compounds α-tomatine and dehydrotomatine are synthesized mainly in the fruit and can also be found in aglycone form as tomatidienol and tomatidine. Esculeoside A and B, dehydrotomatoside also are isolated from the fruit (44, 49–51). In *S. melongena*, α-solamargine, α-solanine, solasonine, and solasodine have been reported in fruit, root, and peel (1, 44, 49, 50). In addition, Lelario, De Maria (52) reported presence of solanidenetriol chacotriose, solanidenedio chacotriose, dehydrosolamargine, solanandaine isomer I, II and II, solanandaine, robenoside B, spirosolenol chacotriose, malonyl-solanandaine, solanidatetraenol chacotriose, arudonine, and malonyl-solamargine. Other species of therapeutic interest, such as *S. torvum* have shown the content of solasodine, solasonine, and solamargine (53–56). Likewise, S. *nigrum* presents solanine and other steroidal glycoalkaloids, such as solasodine, α and β-solamargine, β_2_-solasonine, solasonine, solamargine, and 12β, 27-dihydroxy solasodine (57–59).

### Saponins

Saponins are glycosidic compounds consisting of an aglycone (sapogenin) linked to one or more o oligosaccharide moieties. Saponins are classified according to their structure into steroidal or triterpenoid (60, 61). Most saponins are poorly absorbed in the intestine, have foaming properties in aqueous solutions, exert a hemolytic effect, and cause a bitter taste and astringency (60). Nonetheless, they have been proved to have potential health benefits with anti-insulin resistance and anti-obesogenic effects (62). Saponins obtained from *Solanum* species have shown antitumor, anti-inflammatory, antiviral, antimycotic, antioxidant, hypoglycemic, and hypolipidemic activities (63). In *S. melongena*, the presence of saponins has been reported especially in the seeds; cholestane-type steroidal melongosides-N, O, P, R, S (64), as well as furostanol-type steroidal saponins, melongoside T-V (63). Saponins isolated from *S. melongena* peels showed inhibition of the enzyme lipase, which was more effective than the drug used as control (orlistat) (8). Diosgenin is present in dietary *Solanum* species and has been isolated to study its health-promoting effects: modulating oxidative stress, improving lipid profile, and regulating mitochondrial dysfunction pathway (65). In *S. surattense* at least 11 different saponins have been isolated and shown *in vitro* cytotoxic activities against cancer lines (66). The fruits from *S. torvum* have various steroidal saponins, proven to have anticancer effects against breast, liver, gastric, and lung cancer lines (9, 67).

### Carotenoids

Carotenoids are lipophilic isoprenoid compounds, classified as cyclic or acyclic according to the presence or absence of an end ring on their structure (68–71). The most common carotenoids found in blood plasma are lycopene, β-carotene, and lutein (72). Several health benefits have been attributed to carotenoids like immunomodulators (73, 74), improving eye (75, 76), heart health (77), protecting skin from UV damage, improving brain functions during childhood (78), cancer prevention (79), among others (69). In the *Solanum* genus, *S. lycopersicum* has been an outstanding source of carotenoids, lycopene being the most common (2, 70). Thus, the tomato fruit and its products are the principal sources of lycopene for humans (71). Other types of carotenoids are found in different tomato cultivars are β-carotene, phytoene, and phytofluene (80–82). *S. phureja* has zeaxanthin, violaxanthin, antheraxanthin, lutein, and β-carotene (83). A worldwide important crop *S. tuberosum* is a great source of β-carotene, astaxanthin, and zeaxanthin (71). Less common sources of carotenoids have been found, for example, *S. betaceum* (yellow tamarillo) and *S. sessiliflorum* (cocona). The yellow tamarillo is known for its carotenoid content, being β-cryptoxanthin, β-carotene, zeaxanthin, and lutein, the most abundant (84). Cocona is rich in lycopene and presents higher levels of β-carotene than tomato (85). Carotenoids are pro-vitamin A compounds, a key vitamin for growing, seeing, protecting against diseases, and for reproduction (2).

## Bioaccessibility of Antioxidants in *Solanum* Edibles

It is important to consider the structural changes antioxidants suffer after ingestion. Processing is a starting point in this journey, from cutting or mashing to boiling, baking, or even freezing, that can help release antioxidant compounds, thus increasing their bioaccessibility. Cooking in water can reduce the content of hydrosoluble compounds but cooking with oil can cause synergy and increase the bioaccessibility of lipophilic antioxidants. High temperatures break down cell walls and could increase phytochemicals bioaccessibility, but thermolabile compounds are highly degraded (86). Bioaccessibility is studied using different models to simulate gastrointestinal digestion, allowing researchers to calculate the portion of phytochemicals available for absorption. Phytochemicals can be released from the food matrix due to the simulated conditions based on the physiological data of digestion: electrolytes, digestive enzymes, dilution, pH, and time of digestion (87). Therefore, gastrointestinal digestion can degrade and transform antioxidant compounds, especially those that are highly sensitive to pH changes, such as anthocyanins. Digestive enzymes can also breakdown phytochemicals; like phenolic compounds attached to sugars. Enzymes like the lactase phloridzin hydrolase can hydrolysate sugar moieties from glycosylated bioactive molecules (5, 88).

Eggplant total phenolic content (TPC) was reported before and after four different cooking methods: baking, boiling, frying, and grilling. Antioxidant activity was determined using the ABTS assay, and reducing capacity was quantified using the FRAP (ferric reducing/antioxidant power) assay. Results indicated that TPC was improved >300% by frying, 67% by baking, and 42% by boiling. Grilling eggplant decreased TPC by 34.5%. Raw, boiled, and baked eggplant samples subjected to *in vitro* digestion showed bioaccessibility of TPC of 112.5, 93.4, and 101.8%, respectively; this suggests that phenolic compounds were released after *in vitro* digestion. Fried undigested samples showed the highest amount of TPC, but once the simulated digestion was performed, the bioaccessibility was 67%, the lowest compared to the other three samples. Grilling and *in vitro* digesting eggplant showed that bioaccessibility of TPC went up to 217.4%. The ABTS and FRAP results were consistent with the TPC in all digested and undigested samples. There were two exceptions: the digested boiled samples in the ABTS assay and the raw samples in the FRAP assay. The boiled digested samples increased by 336.4 %; this could be because the phenolic compounds reactive to the ABTS radical were released only after the simulated digestion. Moreover, in the second case, a bioaccessibility of 24% was attributed to a failure in extracting the reducing compounds during digestion, assuming the solvent used with the raw samples was more efficient than *in vitro* digestion (35). Thus, it is suggested that cooking can break down cell walls releasing antioxidants and can increase their bioaccessibility; however, they can also be more susceptible to degradation.

Drying methods have been assessed to preserve the phenolic content and the antioxidant capacity of *S. melongena*. Freezing, drying tunnel, and drying oven methods were evaluated, combined with slicing and mincing the eggplant. Results indicate that sliced eggplant dried at 45–50°C in a drying oven was the best option to obtain a flour rich in bioactive compounds. In the same study, freezing the material had a negative effect on this objective (89). A study was conducted using tomato products to determine the bioaccessibility of lycopene. The tomato pulp was processed by high-pressure homogenization and microwave heating into different end-products in the presence of three different oils: coconut, olive, and fish. High-pressure homogenization, followed by heating at 90°C, increased the bioaccessibility of lycopene. It hypothesized that these processes damage the cellular barriers allowing lycopene to be more bioaccessible (90).

The bioaccessibility of β-carotene in grape tomatoes ranged between 14 and 31%, considering they used two different digestion methods (91). Common home processes like paste processing and drying significantly increased total lycopene, phenolic, and flavonoid content, as well as the total antioxidant capacity. It is suggested that thermal processing disrupts cell membranes and cell walls, releasing lycopene from the insoluble portion of the matrix (92). Synergic interactions have been identified to benefit bioaccessibility. For instance, red cabbage was co-digested with different vegetables, enhancing bioaccessibility of total anthocyanins by 10–15% when samples were carotenoid-rich, like tomatoes. In contrast, the carotenoid bioaccessibility was decreased by 42–56%. This example of phytochemical interaction shows that some combinations exert synergy and others antagonism in bioaccessibility (93). Anthocyanins are highly sensitive to pH changes that naturally occur in the digestive tract, which has led to developing techniques to improve bioaccessibility and bioactivity, such as microencapsulation (94).

After boiling, the bioaccessibility of polyphenols in white and purple potatoes was evaluated. All polyphenols in the samples increased during the gastric phase of the *in vitro* digestion but decreased during the intestinal phase. Nonetheless, the boiled undigested samples had lower content of polyphenols. It is discussed that common chemical extractions underestimate the polyphenol content that could be released in the intestine. p-Coumaric acid in the purple potato was not detectable in the gastric phase, but it was detected in the intestinal phase, and it was 16x higher than in the boiled undigested samples. Also, caffeic acid in white potato was the only phenolic that increased its bioaccessibility in the intestinal phase. It is hypothesized that soluble polyphenols accumulate in cell vacuoles, which are released by pepsin action during gastric digestion. Chlorogenic acid interacts with starch, increasing its bioaccessibility and delaying absorption because it is only released once digested (95).

Two purple potatoes (Amachi and Leona) were subjected to simulated digestion, showing that the total anthocyanin concentration was over 30-fold higher in Amachi compared to Leona digests. In descending colon digesta, concentrations in Leona were 7-fold higher than in Amachi. This data was relevant because of the interest in testing anthocyanins against tumorigenic colon cells (Caco-2). Amachi digesta caused cytotoxicity in non-tumorigenic cells, while Leona's only caused cytotoxicity in tumorigenic cells. Also, it is suggested that microbial metabolism can decrease anthocyanin levels (96).

The bioaccessibility of phenolic compounds in *S. nigrum* leaves to evaluate the effect of heating in the release of phenolic compounds has been studied. The phenolic compounds myricetin, quercetin-3-O-robinoside, 3,4-dicaffeoylquinic acid, 3-caffeoylquinic acid, and rutin were the most abundant. The boiling process improved the phenolic content but decreased after the *in vitro* digestion (36). These results differ from other species of the genus where phenolics were also put through simulated digestion, but the process increases the content. The discrepancies are attributed to the interaction of certain phenolics like chlorogenic acid with starch, which was not abundant in the samples (95). Despite the decreased content of phenolic compounds in *S. nigrum* samples after digestion, they still had bioactivity against oxidative stress and prevented DNA oxidative damage (36).

The fruits of *S. betaceum* are known as a functional ingredient; their phytochemicals have been linked to metabolic syndrome prevention. In this subject, simulated digestion was carried out using the fruit's seeds, pulp, and skin, and bioactive effects such as enzyme inhibition and antioxidant activity were analyzed. Phenolic acids, anthocyanins, condensed tannins, carotenoids (only in pulp) were quantified and showed enzyme inhibitory properties. The extracts were able to inhibit α-glucosidase, α-amylase, and lipase before and after digestion, and the seeds extract inhibition power was improved after digestion. The seed extracts showed to be the most bioactive, and this was linked to the presence of condensed tannins that was higher than in skin and pulp samples (97).

## Implications of the Bioavailability of *Solanum* Antioxidants and their Health-Promoting Properties

The bioavailability of most compounds is crucial to the bioactive effect, and is affected by factors like the individual factors and characteristics of the consumer, interaction with other compounds, delivery matrix, preparation processes, bioactive type/category, chemical structure, and others (98, 99). The pharmacological concept of bioavailability considers liberation, absorption, distribution, metabolism, and excretion, generally known as LADME (100). For all of these factors and the low absorption in the gastrointestinal tract, phytochemicals have low bioavailability (5). Information about the bioavailability of specific phytochemicals is still limited, but we can predict the bioavailability of phytochemicals by using the Lipinski's rule (101); which predicts the drug-likeness of the passive absorption of a molecule considering five chemical characteristics: a molecular weight ≤ 500, partition coefficient (LogP) <5, hydrogen bond donors <5, and a maximum of 10 hydrogen receptors (102) (See Supplementary Table 1).

The evaluation of the bioavailability of phenolic compounds has led to determine that their glycosylation directs the route of their absorption; glycones are transported through active absorption and aglycones through passive diffusion. After their absorption in the intestine, most phenolics are extensively metabolized by xenobiotic enzymes abundant in enterocytes and liver for their excretion through bile, feces, and urine (5). Even though most phenolics are easily degraded during their passage through the gastrointestinal tract, some are highly absorbable. Caffeic acid is 95% absorbed in the small intestine and stomach. Also, chlorogenic acid, the caffeic acid ester form, is absorbed in the colon after the microbiota transforms it into several metabolites (103, 104). The fact that chlorogenic acid usually reaches the colon has been considered beneficial for gut health because it promotes the growth of *Bifidobacterium* species (105). Therefore, chlorogenic acid is considered highly bioavailable for humans; it is estimated that 33% is absorbed intact in the stomach and 7% in the small intestine after hydrolysis (106).

Alkaloids found in *Solanum* have low bioavailability. Therefore, some studies have been focused to improve their absorption, mainly delivery via liposomes, nanoparticles, gels, and emulsions. Transdermal delivery has also been another option, seeking to obtain effective products and avoiding side effects (2, 107). Furthermore, most saponins are hydrosoluble due to their glycosidic groups. They have an amphiphilic nature and, therefore, the ability for self-micellization in the gastrointestinal environment and have shown to be stable to pH variations during digestion. Some saponins can be chemically hydrolyzed by acid or alkali, forming sapogenins, prosapogenins, sugar residues, or monosaccharides. When gastric digestion is simulated, some saponins show deglycosylation, dehydration, hydration, and oxygenation, leading to the presence of different structures connected to saponins' anticarcinogenic activities. Nevertheless, the bioavailability of saponins has not been widely studied (61, 104, 108). Moreover, carotenoids found in cultivars of tomato and their products have been widely studied, and there are valuable results in this context (See Table 2). Pro-vitamin A (β-carotene) has also been evaluated in eggplant. Although it is hypothesized that bioavailability can be reduced when these compounds interact with some vitamins, aspirin, and sulphonamides (69, 113), because these groups of phytochemicals may compete for absorption; for example, co-consumption of lutein has a negative effect on the absorption of β-carotene and vice versa (99, 115) (Table 2).

**Table 2:** Bioavailability of compounds in *Solanum* species.

			**Bioavailability**		
***Solanum* species**	**Material**	**Compounds**	**Intake**	**Final concentration**	**References**
*S. lycopersicum*	Tangerine tomato juice (higher in *cis* lycopene)	Lycopene	10 mg	690.9 nmol h /L	(109)
	Red tomato juice /higher in *trans* lycopene)		10 mg	81.6 nmol h /L	
	Tomato paste		23.6 mg	35.6 nmol/L	(110)
	Fresh tomato		22.2 mg	18.8 nmol h /L	
	Tangerine sauce		13 mg	870.2 nmol h /L	(111)
		B-carotene	17 mg	304 nmol h /L	
	Leaves juice	Pro-vitamin A	0.33 mg		(112)
*S. melongena*	Leaves juice		0.25 mg		
*S. xanthocarpum*	Fruit	β-carotene	45 mg	78–l.23 μmol^*^h /L.	(113)
*S. lycopersicum*	Fresh tomato	β-carotene	2.35 mg	62 nmol^*^h/L	(114)
		Lycopene	13 mg	58 nmol^*^h/L	

* *“per” 78-l.23 μmol “per” h “per” liter*.

## Conclusions

Plants of the *Solanum* genus contain bioactive compounds that are antioxidant agents and have different mechanisms of action to prevent or lessen diseases and their complications. The bioactive potential of diverse materials has been proven, but there is constant interest in evaluating the transformations during their digestion and absorption. The amount of compound or mixture of compounds needed to achieve the desired effect is also a matter of research to formulate effective and safe phytopharmaceuticals. Fruits, roots, and aerial parts of plants among the *Solanum* genus can benefit human beings by improving their health when consumed as part of the daily diet, as a nutraceutical, or biopharmaceutical.

## Author Contributions

EG-G and JH contributed to the conception of the manuscript. CE-R, LC-A, and LM-I contributed to the writing. EG-G and JH edited the manuscript. All authors contributed to the article and approved the submitted version.

## Conflict of Interest

The authors declare that the research was conducted in the absence of any commercial or financial relationships that could be construed as a potential conflict of interest.

## Publisher's Note

All claims expressed in this article are solely those of the authors and do not necessarily represent those of their affiliated organizations, or those of the publisher, the editors and the reviewers. Any product that may be evaluated in this article, or claim that may be made by its manufacturer, is not guaranteed or endorsed by the publisher.
