# Supplementary material for: Solanum Fruits: Phytochemicals, Bioaccessibility and Bioavailability, and Their Relationship With Their Health-Promoting Effects
Source: Front Nutr. 2021 Nov 25;8:790582. doi: 10.3389/fnut.2021.790582 (PMC8687741; doi:10.3389/fnut.2021.790582)
Supplement: Supplementary file 1 [file Table_1.DOCX]

Supplementary Table 1. Predicted passive absorption of some phytochemicals found in Solanum plants.

| Phytochemical | Molecular weight | LogP* | Hydrogen receptors | Hydrogen donors | Predicted absorption |
| --- | --- | --- | --- | --- | --- |
| Diosgenin | 414.6 | 4.93 | 3 | 1 | Yes |
| Melongoside-N | 921.07 | -1.1 | 19 | 12 | No |
| Zeaxanthin | 568.8 | 8.35 | 2 | 2 | No |
| Violaxanthin | 600.8 | 7.26 | 4 | 2 | No |
| α-Solanine | 868.05 | -0.43 | 16 | 19 | No |
| Delphinidin | 303.2 | 2.77 | 7 | 6 | No |
| α-Chaconine | 851.5 | 0.61 | 15 | 8 | No |
| Quercetin | 302.2 | 2.16 | 7 | 5 | Yes |
| Chlorogenic acid | 354.3 | -0.27 | 8 | 6 | No |

*LogP is defined as the partition coefficient. Chemical data obtained from Kim, Chen (1), Wishart, Feunang (2), Wishart, Jewison (3), Wishart, Knox (4), Wishart, Tzur (5)

1. Kim S, Chen J, Cheng T, Gindulyte A, He J, He S, et al. PubChem in 2021: new data content and improved web interfaces. Nucleic Acids Res. 2021;49(D1):D1388-D95.

2. Wishart DS, Feunang YD, Marcu A, Guo AC, Liang K, Vázquez-Fresno R, et al. HMDB 4.0: the human metabolome database for 2018. Nucleic Acids Res. 2018;46(D1):D608-D17.

3. Wishart DS, Jewison T, Guo AC, Wilson M, Knox C, Liu Y, et al. HMDB 3.0—The Human Metabolome Database in 2013. Nucleic Acids Res. 2013;41(D1):D801-D7.

4. Wishart DS, Knox C, Guo AC, Eisner R, Young N, Gautam B, et al. HMDB: a knowledgebase for the human metabolome. Nucleic Acids Res. 2009;37(suppl_1):D603-D10.

5. Wishart DS, Tzur D, Knox C, Eisner R, Guo AC, Young N, et al. HMDB: the Human Metabolome Database. Nucleic Acids Res. 2007;35(suppl_1):D521-D6.
