# Supplementary material for: Solanum Fruits: Phytochemicals, Bioaccessibility and Bioavailability, and Their Relationship With Their Health-Promoting Effects
Source: Front Nutr. 2021 Nov 25;8:790582. doi: 10.3389/fnut.2021.790582 (PMC8687741; doi:10.3389/fnut.2021.790582)
Supplement: Supplementary file 2 [file Presentation_1.pptx]

## Slide 1
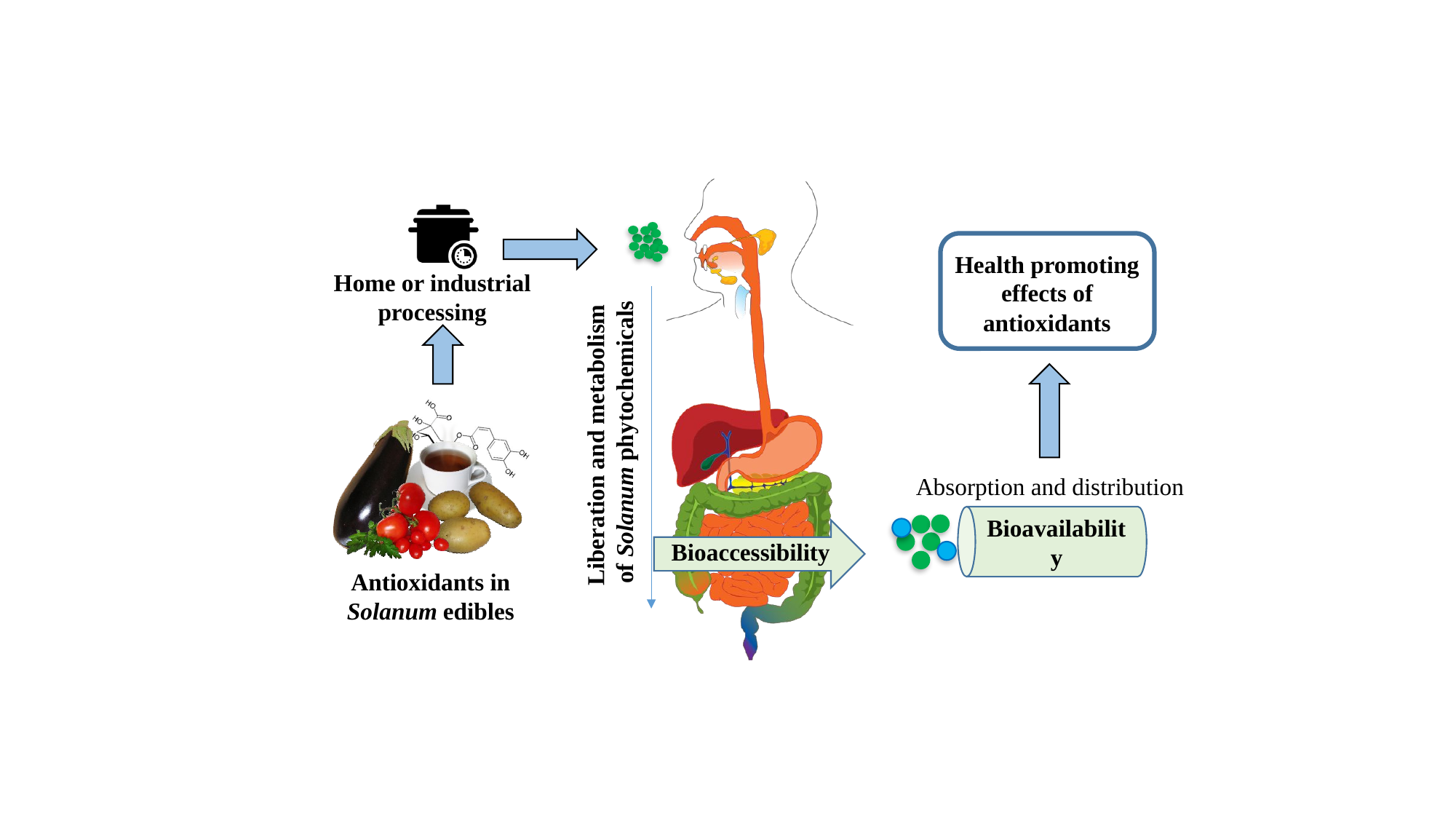

Health promoting effects of antioxidants
Home or industrial processing
Liberation and metabolism
of Solanum phytochemicals
Bioavailability
Bioaccessibility
Absorption and distribution
Antioxidants in Solanum edibles
